# Trace Metals in Vegetables and Associated Health Risks in Industrial Areas of Savar, Bangladesh

**DOI:** 10.5696/2156-9614-10.27.200905

**Published:** 2020-08-19

**Authors:** Md. Al Amin, Md. Estiar Rahman, Sahadat Hossain, Mahmudur Rahman, Mohammad Moshiur Rahman, Md. Jakariya, Md. Tajuddin Sikder

**Affiliations:** 1 Department of Public Health and Informatics, Jahangirnagar University, Savar, Dhaka, Bangladesh; 2 Department of Environmental Science and Management, North South University, Dhaka, Bangladesh

**Keywords:** trace metals, vegetables, health risks, Bangladesh

## Abstract

**Background.:**

The occurrence of high levels of trace metals in foodstuffs represents a significant threat to human health. Vegetables grown in metal-contaminated soil or irrigated with wastewater can accumulate metals and bioaccumulate in the food chain affecting animals and humans.

**Objectives.:**

The present study aimed to measure the levels of lead (Pb), cadmium (Cd), chromium (Cr) and cobalt (Co) in common vegetables grown in the industrial areas of Savar, Bangladesh, and to determine their potential health risks.

**Methods.:**

Five vegetables species: jute (*Corchorus capsularis*), red amaranth (*Amaranthus gangeticus*), okra (*Abelmoschus esculentus*), zucchini (*Luffa aegyptiaca*) and stem amaranth (*Amaranthus viridis*) were sampled randomly from agricultural fields across each study site. Vegetable samples were digested in a microwave digestion system (Berghof Microwave MWS-2, Germany). Metal concentrations were determined using an atomic absorption spectrophotometer (AA-7000, Shimadzu Corporation, Japan).

**Results.:**

The range of Pb, Cd, Cr and Co in analyzed vegetables was 0.643–3.362, 0.041–0.049, 1.681–2.431 and 1.612–2.492 mg/kg, respectively. The target hazard quotient (THQ) of Pb in zucchini and stem amaranth and the THQs of Cr in all analyzed vegetables was greater than one. The target carcinogenic risk (TCR) of Pb and Cd for all analyzed vegetables was in the unacceptable range. In all vegetable samples, lead content was detected to be higher than the maximum permissible limits. The THQ values indicate the possibility of non-carcinogenic health risk through consumption of these vegetables. In addition, the TCR values of Pb and Cd indicate a lifetime carcinogenic health risks to consumers.

**Conclusions.:**

Consumption of vegetables grown in this area may pose long-term health risks.

**Competing Interests.:**

The authors declare no competing financial interests.

## Introduction

Trace metals are widely distributed in the environment. In addition to their vital role in plant growth and/or human nutrition, some metals, such as arsenic (As), lead (Pb), cadmium (Cd), cobalt (Co), nickel (Ni), chromium (Cr), mercury (Hg) etc., cause adverse effects to human health even at low concentrations.^1^ The occurrence of metals in food can have geogenic or anthropogenic origins.^2^ Evidence suggests that anthropogenic activities such as wastewater irrigation, solid waste disposal, mining, smelting, sewage sludge applications, fertilizers, fungicides and industrial activities contribute significantly to the deposition of these metals in cultivable land.^3^–^5^ Crops and vegetables grown in contaminated soil or irrigated with wastewater could take up metals and accumulate them in their edible parts.^6^ Human can be exposed to metals through various pathways. One major exposure pathway is through consumption/diet.^4^ Vegetables constitute an important part of the human diet. Therefore, it is reasonable to hypothesize that vegetables contaminated with metals pose a potential health risk to consumers, in addition to their ability to fulfill nutritional requirements.

Some metals like copper (Cu), zinc (Zn), selenium (Se), etc. are essential for maintaining the physiological functions and biochemical processes in humans.^7^ However, metals like As, Pb, Cd, Co, Cr, Hg are considered potentially toxic because these metals cause adverse effects to human health when ingested over a long period.^8^,^9^ Moreover, metals like As, Pb and Cd have shown carcinogenic effects.^10^ Previous studies have reported the concentration of metals in different foodstuffs.^11^–^15^ Vegetables can uptake metals from polluted soil and water, which depends largely on soil composition, water quality, metal solubility, and absorption ability.^6^,^11^ The exceedingly higher concentrations of metals in vegetables pose serious health risks to humans through the food chain.^1^,^16^

Over the past decades, Bangladesh has undergone structural transformation from agriculture to industrialization. Studies show that vegetables growing near industrial zones display higher concentration of metals.^11^,^16^ Savar upazila is an important industrial zone in Bangladesh. Most of its industries are located at the bank of the Turag River. Several studies have reported on concentrations of metals in this river's water.^17^,^18^ Moreover, the area is exposed to different degrees of industrial pollution, especially from tannery waste and textile dyeing. The vegetables growing in this area may become contaminated by metals through water irrigation or soil absorption. Previous studies revealed metals like As, Cd, Pb, Hg, Cr, manganese (Mn), Ni, Cu, and Zn are significant vegetable contaminants in Bangladesh.^11^,^12^,^19^ However, the potential health risk associated with the consumption of vegetables in the study area of Bangladesh has not been fully investigated. Therefore, the present study aimed to measure the levels of Pb, Cd, Cr and Co in commonly consumed vegetables grown in industrial areas of Savar upazila, Bangladesh, and to determine associated potential health risks. In the present study, health risks from exposure to Pb, Cd, Cr and Co through the consumption of vegetables were evaluated using different indices, including estimated daily intake of metals (EDI), target hazard quotient (THQ), hazard index (HI) and target carcinogenic risk (TCR). The THQ and HI evaluate potential non-carcinogenic risk, while TCR evaluates carcinogenic risk that can occur due to consumption of these vegetables.

**Abbreviations**
*EDI*: Estimated daily intake
*fw*: Fresh weight
*HI*: Hazard index
*TCR*: Target carcinogenic risk
*THQ*: Target hazard quotient

## Methods

**Figure 1:**
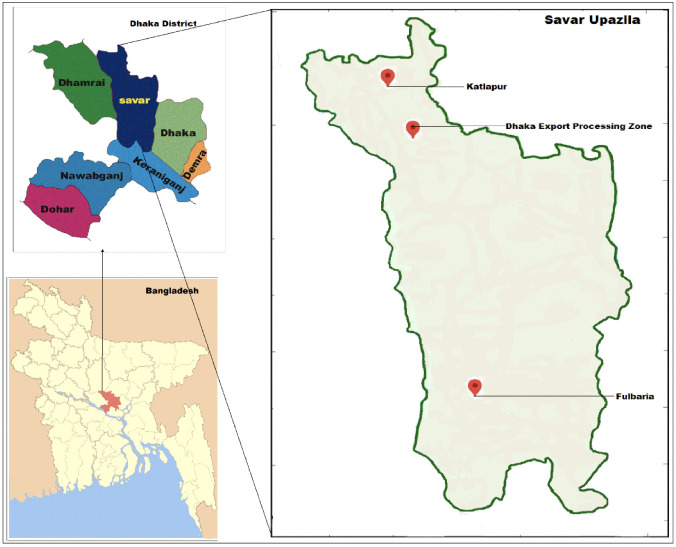
Sampling sites of the study area (Savar)

The samples were collected from Fulbaria, Katlapur, and the Dhaka Export Processing Zone of Savar upazila *(Figure 1).* The area of Savar upazila is 280.13 km^2^ and located at 23.8583° N 90.2667° E. It is situated about 25 km to the northwest of Dhaka city (the capital city of Bangladesh). Savar is an important industrial region of Bangladesh and is highly vulnerable to environmental pollution. The area is home to a variety of industries such as garments, tanneries, pharmaceuticals, packaging, dyeing, battery manufacturing, textile, and different food processing industries which produce large volumes of effluents that may contain heavy metals. After production, these industries discharge untreated wastes randomly into nearby rivers and canals. The disposed wastes are then mixed with soils, which are continuously being polluted by toxic elements in the area.

### Sample collection and preparation

Five commonly consumed and seasonally available vegetables species, i.e. jute *(Corchorus capsulari*s), red amaranth *(Amaranthus gangeticus)*, okra *(Abelmoschus esculentus),* zucchini *(Luffa aegyptiaca)* and stem amaranth *(Amaranthus viridis)* were collected randomly from agricultural fields across each site between May and August in 2019 *(Table 1).* The samples were collected by hand using vinyl gloves, carefully packed into polyethylene bags, and then brought to the laboratory for analysis. The analysis was done at the Wazed Miah Science Research Center, Jahangirnagar University. Only the edible parts of the collected vegetable samples were used. The samples were washed with distilled water to remove foreign materials. The samples were partially dried at room temperature to remove moisture and then cut into small pieces with a knife of stainless steel. The samples were then oven-dried at 80°C for 72 hours. The oven dried samples were powdered using a pestle and mortar and sieved through a mesh of 2 mm.

**Table 1:** Description of Collected and Analyzed Vegetable Samples

**Local name (Bangla)**	**English name**	**Scientific name**	**Edible parts**
Pat shak	Jute	*Corchorus capsularis*	Leaf
Lal shak	Red amaranth	*Amaranthus gangeticus*	Leaf and stem
Dheros	Okra	*Abelmoschus esculentus*	Fruit
Dhundul	Zucchini	*Luffa aegyptiaca*	Fruit
Data shak	Stem amaranth	*Amaranthus viridis*	Stem

### Metal analysis

Analytical reagent grade chemicals and deionized water were used to prepare all solutions. A total of 0.5 g of each powdered sample was treated with 65% nitric acid and 30% hydrochloric acid in the volume ratio of 5:1 in a closed Teflon vessel and then digested in a microwave digestion system (Berghof Microwave MWS-2, Germany). Samples were cooled at room temperature and filtered using Whatman filter paper. The final volume was made up to 50 ml using double distilled water. Metal concentrations were determined using an atomic absorption spectrophotometer (AA-7000, Shimadzu Corporation, Japan). Standard solution (1000 mg/l) of different metals viz. Pb, Cd, Cr and Co were procured from Agilent Technologies. A standard curve was prepared using various concentrations made from standard solution. Digested samples were then analyzed for the metal content. All test batches were evaluated using an internal quality approach and validated if they satisfied the defined internal quality controls. Blank samples were scrutinized after every three samples for the purpose of ensuring that obtained results were within the correct range. The levels of metals were calculated based on dry weight and all the examinations were replicated in triplicate.

### Estimated daily intake of metals

The EDI of metals via consumption of foods depends on the metal concentrations in foods, daily food consumption, as well as body weight. In the present study, the EDI of the metals of interest (Pb, Cd, Cr and Co) were evaluated with Equation 1.^20^

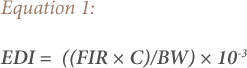



In Equation 1, FIR is the daily vegetable consumption rate (g/person/day), C is the estimated metal concentration in vegetable samples (mg/kg fresh weight (fw)), BW is the average body weight and 10^−3^ is the conversion factor *(Table 2).*

**Table 2:** Input Parameters to Characterize the Estimated Daily Intake, Target Hazard Quotient and Target Carcinogenic Risk

**Exposure parameters**	**Symbols**	**Units**	**Values**	**References**
**Metal concentration**	C	mg/L	Table 3	
**Ingestion rate**	F_IR_	kg/day	^a^	21
**Exposure frequency**	EF	days/year	365	22
**Exposure duration**	ED	Years	^b^	7
**Average exposure time**	AT	Days	^c^	8
**Body weight**	BW	Kg	^d^	7
**Oral reference rose**	RFD	mg/kg/day	^e^	23,24
**Cancer slope factor**	CSF	mg/kg/day	^f^	7

^a^ Ingestion rate of vegetables for adults (154.95 kg/day) and for children (105.6 g/day).

^b^ Exposure duration for adults (70 years) and for children (10 years).

^c^ Average exposure time to the toxicants (365 days × 70 years).

^d^ Body weight for adults (72 kg) and for children (32.7 kg).

^e^ Oral reference dose (0.004, 0.001, 0.003, and 0.03 mg/kg/day for Pb, Cd, Cr and Co, respectively)

^f^ Cancer slope factor (0.0085 and 3.8 mg/kg/day for Pb and Cd, respectively).

### Target hazard quotient

The THQ can be defined as the ratio of exposure to a toxic substance and the level at which no adverse health effects are expected. A THQ value less than one implies no non-carcinogenic health risk from lifetime exposure to the substance, while if the THQ is higher than one, the toxicant may produce an adverse non-carcinogenic effect. The equation used for estimating the THQ is as follows.^20^

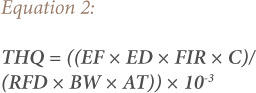



In Equation 2, EF is the exposure frequency (365 days/year), ED is the exposure duration, FIR is the daily vegetable consumption rate (g/person/day), C is the estimated metal concentration in vegetable samples (mg/kg fw), RFD is the oral reference dose (mg/kg/day), BW is the average body weight, AT is the average exposure time for non-carcinogenic effects and 10^−3^ is the conversion factor. The main exposure factors that have been taken into account to carry out the risk assessment calculations are listed in Table 2.

### Hazard index

The HI is the sum of the THQs of individual metals in each food item. The HI assumes that exposure to multiple metals results in additive effects. Even if the individual THQs for the metals in the food item are lower than one individually, adverse health effects may be posed by the cumulative effect of consumption. A hazard index greater than one indicates significant non-carcinogenic risks.^25^ The following equation was used for HI evaluation.^20^

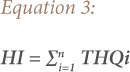



### Target carcinogenic risk

The TCR is the possibility of an individual lifetime health risk of developing cancer upon exposure to carcinogens. A TCR value between 10^−6^ and 10^−4^ indicates an increased risk of developing cancer. A value of 10^−4^ is the upper limit of the range and a value above this range indicates lifetime cancer risk to populations who consumed contaminated foods.^26^ Lead and Cd are considered carcinogenic elements.^10^ The carcinogenic potential of these metals was calculated using the following equation:^20^

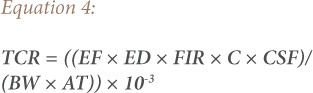
In Equation 4, EF is the exposure frequency (365 days/year), ED is the exposure duration, FIR is the daily vegetable consumption rate (g/person/day), C is the estimated metal concentration in vegetable samples (mg/kg fw), CSF is the oral carcinogenic slope factor (mg/kg/day), BW is the average body weight, AT is the average exposure time for non-carcinogenic effects and 10^−3^ is the conversion factor. The oral carcinogenic slope factors for Pb and Cd are presented in Table 2.

### Data analysis

All the calculations were performed using Microsoft Office Excel 2019. Descriptive statistics such as mean, SD, and ranges for the target parameters were calculated. Tables were used to describe the calculations.

## Results

The concentrations of Pb, Cd, Cr and Co in five vegetable species, including jute *(Corchorus capsulari*s), red amaranth *(Amaranthus gangeticus)*, okra *(Abelmoschus esculentus),* zucchini *(Luffa aegyptiaca)* and stem amaranth *(Amaranthus viridis)* grown in the industrial areas of Savar upazila, Bangladesh are presented in Table 3. All results are reported as fresh weight (fw). Of these analyzed vegetable species, okra had the lowest mean concentration of Pb at 0.643 mg/kg and zucchini had the highest mean concentration of Pb at 3.362 mg/kg. The lowest Cd content was 0.041 mg/kg detected in zucchini, while the highest Cd content (0.049 mg/kg) was detected in okra. The results of this study showed that the Cr content ranged from 1.681 mg/kg detected in stem amaranth to a high of 2.431 mg/kg in red amaranth. The Co content was the lowest in zucchini (1.612 mg/kg) and highest in red amaranth (2.492 mg/kg).

**Table 3:** Estimated Metal Content (mg/kg fw) in Analyzed Vegetables

**Samples**	**Statistics**	**Pb**	**Cd**	**Cr**	**Co**
Jute	Mean ± SD	0.697±0.26	0.044±0.005	2.182±0.49	2.231±0.56
	Range	0.45–0.97	0.04–0.049	1.64–2.61	1.58–2.58
Red amaranth	Mean ± SD	0.721±0.43	0.046±0.003	2.431±0.63	2.492±0.47
	Range	0.26–1.12	0.0427–0.049	1.72–2.89	2.05–2.45
Zucchini	Mean ± SD	3.362±0.5	0.041±0.007	1.842±0.63	1.612±0.49
	Range	2.78–3.67	0.0338–0.049	1.12–2.21	1.21–2.16
Okra	Mean ± SD	0.643±0.28	0.049±0.004	2.193±0.57	1.761±0.44
	Range	0.39–0.95	0.0445–0.053	1.85–2.85	1.27–2.12
Stem amaranth	Mean ± SD	1.951±1.45	0.042±0.005	1.681±0.27	2.332±0.48
	Range	0.69–3.59	0.038–0.048	1.50–1.99	2.02–2.09
MAC^27^		0.10	0.05	2.30	50.00

Abbreviation: MAC, maximum allowable concentration (mg/kg fw).

### Estimated daily intake of heavy metals

The EDIs of four metals (Pb, Cd, Cr and Co) were calculated with Equation 1 and are presented in Table 4. Maximum tolerable daily intake of these corresponding metals is also presented. Currently no maximum tolerable daily intake value exists for Co. In vegetable samples, mean values of EDI decreased in the following order: Cr > Co > Pb > Cd. Total daily intake of Pb, Cd, Cr and Co ranged between [0.002–0.0109], [0.0001–0.0002], [0.0054–0.0079] and [0.0052–0.0081] mg/kg body weight, respectively for children and [0.0014–0.0072], [0.0001–0.0002], [0.0036–0.0052], [0.0035–0.0054] mg/kg body weight, respectively, for adults.

**Table 4:** Estimated Daily Intake of Metals via Consumption of Vegetables

**Samples**	**Individuals**	**EDI (mg/kg body wt/day)**				**Combined metal EDI**

		**Pb**	**Cd**	**Cr**	**Co**	
Jute	Children	0.0023	0.0001	0.0070	0.0072	0.0166
	Adults	0.0015	0.0001	0.0047	0.0048	0.0111
Red amaranth	Children	0.0023	0.0002	0.0079	0.0081	0.0185
	Adults	0.0016	0.0001	0.0052	0.0054	0.0123
Zucchini	Children	0.0109	0.0002	0.0060	0.0052	0.0223
	Adults	0.0072	0.0002	0.0040	0.0035	0.0149
Okra	Children	0.0021	0.0002	0.0071	0.0057	0.0151
	Adults	0.0014	0.0001	0.0047	0.0038	0.0100
Stem amaranth	Children	0.0063	0.0001	0.0054	0.0076	0.0194
	Adults	0.0042	0.0001	0.0036	0.0050	0.0129
MTDI		0.21^28^	0.05^28^	0.20^29^	–	

Abbreviation: MTDI: maximum tolerable daily intake.

### Non-carcinogenic risk

**Figure 2:**
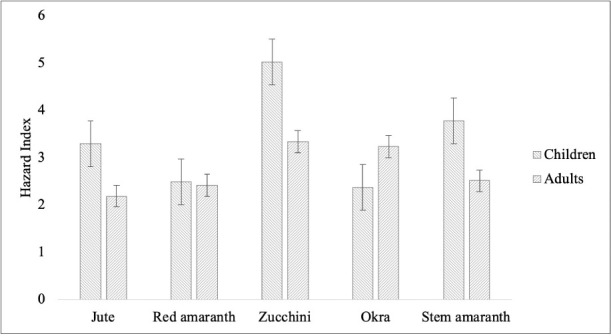
Hazard index for metals in analyzed vegetables

The THQs for non-carcinogenic risk of the tested metals from consumption of vegetables for children and adults are presented in Table 5. The THQ for Pb varied from 0.519 to 2.72, 0.134 to 0.157 for Cd, 1.812 to 2.629 for Cr, and 0.174 to 0.269 for Co for children; while Pb varied from 0.344 to 1.807, 0.089 to 0.105 for Cd, 1.203 to 1.746 for Cr and 0.115 to 0.179 for Co for adults. The findings show that the THQ values of Pb in zucchini and stem amaranth and the THQs of Cr in all analyzed vegetables were greater than one. The HI was calculated to assess multiple effects of metals, as shown in Figure 2. In the present study, the HI for all analyzed vegetable samples was greater than one.

**Table 5:** Target Hazard Quotients for Metals in Analyzed Vegetable Samples

**Vegetable**	**Individuals**	**Target Hazard Quotient**			

		**Pb**	**Cd**	**Cr**	**Co**
Jute	Children	0.565	0.141	2.341	0.241
	Adults	0.375	0.094	1.555	0.160
Red amaranth	Children	0.583	0.150	2.629	0.269
	Adults	0.386	0.100	1.746	0.179
Zucchini	Children	2.720	0.134	1.992	0.174
	Adults	1.807	0.089	1.323	0.115
Okra	Children	0.519	0.157	2.370	0.190
	Adults	0.344	0.105	1.574	0.126
Stem amaranth	Children	1.578	0.137	1.812	0.252
	Adults	1.048	0.091	1.203	0.167

### Carcinogenic risk

The carcinogenic risks of Pb and Cd were evaluated based on the TCR from consumption of vegetables and are presented in Table 6. In this study, the TCR values of Pb ranged from 1.8E-05 in okra to 9.3E-05 in zucchini and 5.1E-04 in zucchini to 5.9E-04 in okra for Cd for children *(Table 6).* For adults, the TCR values of Pb ranged from 1.2E-05 in okra to 6.1E-05 in zucchini and 3.4E-04 in zucchini to 3.9E-04 in okra for Cd.

**Table 6:** Target Carcinogenic Risks of Lead and Cadmium in Analyzed Vegetables

**Samples**	**Individuals**	**TCR**		**Combined metal TCR**

		**Pb**	**Cd**	
Jute	Children	1.9E-05	5.4E-04	5.59E-04
	Adults	1.3E-05	3.6E-04	3.73E-04
Red amaranth	Children	1.9E-05	5.7E-04	5.89E-03
	Adults	1.3E-05	3.7E-04	3.83E-04
Zucchini	Children	9.3E-05	5.1E-04	6,03E-04
	Adults	6.1E-05	3.4E-04	4.01E-04
Okra	Children	1.8E-05	5.9E-04	6.08E-04
	Adults	1.2E-05	3.9E-04	4.02E-04
Stem amaranth	Children	5.4E-05	5.2E-04	5.74E-04
	Adults	3.6E-05	3.5E-04	3.86E-04

## Discussion

The present study aimed to measure the levels of Pb, Cd, Cr and Co in commonly consumed vegetables grown in the industrial areas of Savar, Bangladesh, and to determine their potential health risks. The average levels of metals occurred in the following declining order in all vegetable samples: Co > Cr > Pb > Cd *(Table 3).* A great variety of metal concentrations were observed among different vegetable species. The accumulation of these metals were attributed to the differential absorption capacities of vegetables for different metals.^6^,^13^

Lead, a highly toxic metal, is absorbed in inorganic forms through ingestion of food and water and inhalation. Lead is known to induce kidney damage, increase blood pressure and cardiovascular disease risk for adults and hinder cognitive development in children.^30^ In the present study, Pb content ranged from 0.643 mg/kg in okra to a high of 3.362 mg/kg in zucchini. In the literature, Pb content was reported as 0.692–3.152 mg/kg in vegetable samples along roadsides in Savar.^31^

More recent studies conducted in Bangladesh have reported Pb concentrations of 0.84–28.18 mg/kg, 0.005–0.057 mg/kg, and 1.5–3.5 mg/kg in different vegetable species.^12^,^19^,^23^ The maximum permissible limit of Pb in vegetables on harvesting is 0.1 mg/kg.^27^ In the present study, Pb contents in all analyzed vegetable samples were higher than the recommended permissible limit. The higher level of Pb in vegetable samples may be due to lead smelting activity in the study area. Lead is known to emanate from industrial activities, such as battery production and processing, plastic manufacturing, cement production and processing, fertilizer production, metal workshops, and car repair and welding workshops which can impact vegetables grown in industrial areas.^32^

Cadmium is a metallic element that occurs naturally at low levels in the environment and is toxic even at low concentrations. Cadmium is classified as a human carcinogen and long term exposure exerts toxic effects on the kidney, skeletal and digestive systems.^33^ In the present study, the lowest Cd content was 0.041 mg/kg found in zucchini, while the highest Cd content (0.049 mg/kg) was found in okra. Previous studies from Savar areas showed that Cd content in vegetable samples ranged from 0.18 mg/kg to 2.31 mg/kg, and 1.03 mg/kg to 4.65 mg/kg.^11^,^31^ Recent Bangladeshi studies have reported Cd concentrations in vegetables of 1.39–4.09 mg/kg and 0.008–0.056 mg/kg.^12^,^19^ The mean concentrations of Cd in all vegetable samples in this study were close to the maximum permissible limit (0.05 mg/kg).^27^ Cadmium is a common pollutant present in the soils of industrial areas.^34^ Anthropogenic activities like the textile industry, pharmaceuticals companies, fertilizer application, automobile workshops etc. result in soil contamination with Cd and vegetables grown in the contaminated soil could accumulate the metal.

Chromium is an essential trace mineral that humans need in very small quantities. Chronic exposure to high levels of Cr leads to the development of gastrointestinal and central nervous system cancers.^35^ The results of this study showed that stem amaranth samples had the lowest mean concentration of Cr at 1.681 mg/kg and red amaranth samples had the highest mean concentration of Cr at 2.431 mg/kg. Chromium contents in all studied vegetables (except red amaranth) were found to be below the standard limit (2.3 mg/kg).^27^ In the literature, Cr concentrations of 1.171–3.835 mg/kg have been reported in vegetables in Savar areas, and 2.28–11.84 mg/kg in vegetables of the Dhaka Export Processing Zone areas (also in Savar).^11^,^31^ Previous Bangladeshi studies found Cr concentrations in vegetables of 2.10 mg/kg to 33.16 mg/kg and 0.30 mg/kg to 1.11 mg/kg.^12^,^19^ The occurrence of Cr in vegetables may be attributed to soil uptake. The main sources of Cr in soils are the application of chemical fertilizers and pesticides, wastewater irrigation and open dumping of industrial wastes in the environment.^36^

Cobalt is another essential trace mineral required by humans for proper functioning of bodily systems. However, when ingested beyond permissible limits over a long period of time, Co can lead to human neurological, cardiovascular and endocrine deficits.^37^ In the present study, the Co content was found to be the lowest in zucchini (1.612 mg/kg) and highest in red amaranth (2.492 mg/kg). Mean Co concentrations in all the vegetable samples were below the maximum permissible limit (50 mg/kg).^27^

The EDIs of Pb, Cd and Cr from individual vegetable species were below the maximum tolerable daily intakes recommended by international regulatory bodies; indicating that these vegetable species may not pose health risks to consumers. However, combined metal EDIs might pose a risk to consumers.

In the present study, the non-carcinogenic risk from consuming vegetables in the study area was evaluated for adults and children. The THQ for non-carcinogenic risk of Pb in zucchini and stem amaranth, and the THQs of Cr in all analyzed vegetables were greater than one, indicating a non-carcinogenic health risk through consumption of vegetables. These findings indicate that Pb and Cr were the major components contributing to the potential health risk via consumption of the analyzed vegetables. Therefore, consumers are at high risk with respect to Cr which can pose non-carcinogenic risks to adults and children. The HI assesses multiple effects of metals. The potential risk could be multiplied when considering all the metals together. In the present study, the HI for all analyzed vegetable samples was greater than one, indicating potential non-carcinogenic health risks from consuming analyzed vegetables for both children and adults. Consequently, consumption of these vegetables is considered unsafe and their consumption on regular basis is not recommended. Therefore, consumers (both children and adults) are at high risk with respect to these metals which can cause non-carcinogenic effects.

The present study also evaluated the carcinogenic risk from Pb and Cd for adults and children from consuming vegetables, as Pb and Cd show carcinogenic effects.^10^ The TCRs for carcinogenic risk of Pb for all analyzed vegetables were in the unacceptable range *(Table 6),* and the TCRs of Cd for all analyzed vegetable samples exceed 10^−4^, both for children and adults, indicating potential carcinogenic risks for the consumers of these vegetables. Therefore, the carcinogenic risks posed by these metals to the local residents via consumption of these vegetables should be addressed.

The present study had some limitations. Due to limited resources and financial constraints, we did not measure metals such as As, Hg, Ni, etc. In addition, other exposure pathways for metals such as dermal contact, inhalation, etc. were not investigated. Despite these limitations, this study revealed important findings concerning metal contamination in vegetables and possible risks to human health.

## Conclusions

While further research on human health risks associated with metals in a wider context is needed, this initial study found that vegetables in the Savar industrial area of Bangladesh accumulated Pb, Cd and Cr at high concentrations compared to the maximum permissible limits. The THQ and HI values showed that consumers face non-carcinogenic health risks due to the consumption of metal-contaminated vegetables. The TCR values for Cd exceeded the threshold level, indicating that consumers of these vegetables might face lifelong carcinogenic risk. In this context, based on potential risks to human health, efforts at remediation should include reducing anthropogenic activities that contribute to contamination with these metals.

## References

[1] Jaishankar M, Tseten T, Anbalagan N, Mathew BB, Beeregowda KN (2014). Toxicity, mechanism and health effects of some heavy metals. Interdiscip Toxicol.

[2] Armah FA, Quansah R, Luginaah I (2014). A systematic review of heavy metals of anthropogenic origin in environmental media and biota in the context of gold mining in Ghana. Int Sch Res Notices [Internet].

[3] Mingorance MD, Valdes B, Oliva SR (2007). Strategies of heavy metal uptake by plants growing under industrial emissions. Environ Int [Internet].

[4] Xue ZJ, Liu SQ, Liu YL, Yan YL (2012). Health risk assessment of heavy metals for edible parts of vegetables grown in sewage-irrigated soils in suburbs of Baoding City, China. Environ Monit Assess [Internet].

[5] Laniyan TA, Adewumi AJ (2020). Evaluation of contamination and ecological risk of heavy metals associated with cement production in Ewekoro, Southwest Nigeria. J Health Pollut [Internet].

[6] Agrelli D, Adamo P, Cirillo T, Duri LG, Duro I, Fasano E, Ottaiano L, Ruggiero L, Scognamiglio G, Fagnano M (2017). Soil versus plant as indicators of agroecosystem pollution by potentially toxic elements. J Plant Nutr Soil Sci [Internet].

[7] Mirzabeygi M, Abbasnia A, Yunesian M, Nodehi RN, Yousefi N, Hadi M, Mahvi AH (2017). Heavy metal contamination and health risk assessment in drinking water of Sistan and Baluchistan, Southeastern Iran. Hum Ecol Risk Assess [Internet].

[8] Esposito F, Nardone A, Fasano E, Scognamiglio G, Esposito D, Agrelli D, Ottaiano L, Fagnano M, Adamo P, Beccaloni E, Vanni F, Cirillo T (2018). A systematic risk characterization related to the dietary exposure of the population to potentially toxic elements through the ingestion of fruit and vegetables from a potentially contaminated area. A case study: the issue of the “Land of Fires” area in Campania region, Italy. Environ Pollut [Internet].

[9] Chauhan G, Chauhan U (2014). Human health risk assessment of heavy metals via dietary intake of vegetables grown in wastewater irrigated area of Rewa, India. Int J Sci Res Publ [Internet].

[10] List of classifications by cancer sites with sufficient or limited evidence in humans.

[11] Ahmad JU, Goni MA (2010). Heavy metal contamination in water, soil, and vegetables of the industrial areas in Dhaka, Bangladesh. Environ Monit Assess [Internet].

[12] Proshad R, Kormoker T, Islam MS, Chandra K (2020). Potential health risk of heavy metals via consumption of rice and vegetables grown in the industrial areas of Bangladesh. Hum Ecol Risk Assess [Internet].

[13] Pandey J, Pandey U (2009). Accumulation of heavy metals in dietary vegetables and cultivated soil horizon in organic farming system in relation to atmospheric deposition in a seasonally dry tropical region of India. Environ Monit Assess [Internet].

[14] Mar KM (2020). Cadmium uptake and relationship to feeding habits of freshwater fish from the Ayeyarwady River, Mandalay, Myanmar. J Health Pollut [Internet].

[15] Oladipo OO, Akanbi OB, Ekong PS, Uchendu C, Ajani O (2020). Lead toxicoses in free-range chickens in artisanal gold-mining communities, Zamfara, Nigeria. J Health Pollut [Internet].

[16] Cherfi A, Achour M, Cherfi M, Otmani S, Morsli A (2015). Health risk assessment of heavy metals through consumption of vegetables irrigated with reclaimed urban wastewater in Algeria. Process Saf Environ Prot [Internet].

[17] Sikder T, Yasuda M, Yustiawati, Syawal SM, Saito T, Tanaka S, Kurasaki M (2012). Comparative Assessment on Water Quality in the Major Rivers of Dhaka and West Java. Int J Environ Prot.

[18] Sikder MT, Kihara Y, Yasuda M, Yustiawati, Mihara Y, Tanaka S, Odgerel D, Mijiddorj B, Syawal SM, Hosokawa T, Saito T, Kurasaki M (2013). River water pollution in developed and developing countries: judge and assessment of physicochemical characteristics and selected dissolved metal concentration. Clean Soil Air Water [Internet].

[19] Shaheen N, Irfan NM, Khan IN, Islam S, Islam MS, Ahmed MK (2016). Presence of heavy metals in fruits and vegetables: Health risk implications in Bangladesh. Chemosphere [Internet].

[20] (1989). Assessing human health risks from chemically contaminated fish and shellfish: a guidance manual [Internet]. https://nepis.epa.gov/Exe/ZyPURL.cgi?Dockey=2000DGLF.TXT.

[21] Report of the household income & expenditure survey 2010 [Internet].

[22] Giri S, Singh AK (2015). Human health risk assessment via drinking water pathway due to metal contamination in the groundwater of Subarnarekha River Basin, India. Environ Monit Assess [Internet].

[23] Islam S, Ahmed K, Habibullah-Al-Mamun M, Raknuzzaman M (2015). The concentration, source and potential human health risk of heavy metals in the commonly consumed foods in Bangladesh. Ecotoxicol Environ Saf [Internet].

[24] Finley BL, Monnot AD, Paustenbach DJ, Gaffney SH (2012). Derivation of a chronic oral reference dose for cobalt. Regul Toxicol Pharmacol [Internet].

[25] Kacholi DS, Sahu M (2018). Levels and health risk assessment of heavy metals in soil, water, and vegetables of Dar es Salaam, Tanzania. J Chem [Internet].

[26] Fryer M, Collins CD, Ferrier H, Colvile RN, Nieuwenhuijsen MJ (2006). Human exposure modelling for chemical risk assessment: a review of current approaches and research and policy implications. Environ Sci Policy [Internet].

[27] (2011). Working document for information and use in discussions related to contaminants and toxins in the GSCTFF [Internet]. Joint FAO/WHO Food Standards Programme Codex Committee on Contaminants in Foods, Fifth Session; 2011 Mar 21–25; The Hague.

[28] (2010). Evaluation of certain food additives and contaminants: seventy-third report of the Joint FAO/WHO Expert Committee on Food Additives [Internet]. Seventy-third meeting of the Joint FAO/WHO Expert Committee on Food Additives.

[29] (1989). Recommended dietary allowances.

[30] Assi MA, Hezmee MN, Haron AW, Sabri MY, Rajion MA (2016). The detrimental effects of lead on human and animal health. Vet World.

[31] Aktaruzzaman M, Fakhruddin AN, Chowdhury MA, Fardous Z, Alam MK (2013). Accumulation of heavy metals in soil and their transfer to leafy vegetables in the region of Dhaka Aricha Highway, Savar, Bangladesh. Pak J Biol Sci.

[32] Mitra AK, Haque A, Islam M, Bashar SA (2009). Lead poisoning: an alarming public health problem in Bangladesh. Int J Environ Res Public Health [Internet].

[33] Godt J, Scheidig F, Grosse-Siestrup C, Esche V, Brandenburg P, Reich A, Groneberg DA (2006). The toxicity of cadmium and resulting hazards for human health. J Occup Med Toxicol [Internet].

[34] Mahmud MA, Hassan M, Hassan R, Mandal R, Rahman MK (2017). Human health risk assessment due to Cadmium accumulation through consumption of Chinese cabbage grown in Cadmium-contaminated soil. J Biodivers Conserv Bioresour Manag [Internet].

[35] Costa M, Klein CB (2006). Toxicity and carcinogenicity of chromium compounds in humans. Crit Rev Toxicol [Internet].

[36] Uddin AN, Ahmed SA (2018). Heavy metal contamination of soil and health hazards among the residents of tannery industrial area. Anwer Khan Mod Med Coll J [Internet].

[37] Scharf B, Clement CC, Zolla V, Perino G, Yan B, Elci SG, Purdue E, Goldring S, Macaluso F, Cobelli N, Vachet RW, Santambrogio L (2014). Molecular analysis of chromium and cobalt-related toxicity. Sci Rep [Internet].

